# Quantitative-Proteomic Comparison of Alpha and Beta Cells to Uncover Novel Targets for Lineage Reprogramming

**DOI:** 10.1371/journal.pone.0095194

**Published:** 2014-04-23

**Authors:** Amit Choudhary, Kaihui Hu He, Philipp Mertins, Namrata D. Udeshi, Vlado Dančík, Dina Fomina-Yadlin, Stefan Kubicek, Paul A. Clemons, Stuart L. Schreiber, Steven A. Carr, Bridget K. Wagner

**Affiliations:** 1 Society of Fellows, Harvard University, Cambridge, Massachusetts, United States of America; 2 Center for the Science of Therapeutics, Broad Institute, Cambridge, Massachusetts, United States of America; 3 Department of Molecular and Cellular Biology, Harvard University, Cambridge, Massachusetts, United States of America; 4 Department of Chemistry and Chemical Biology, Harvard University, Cambridge, Massachusetts, United States of America; 5 Howard Hughes Medical Institute, Chevy Chase, Maryland, United States of America; University of Lille Nord de France, France

## Abstract

Type-1 diabetes (T1D) is an autoimmune disease in which insulin-secreting pancreatic beta cells are destroyed by the immune system. An emerging strategy to regenerate beta-cell mass is through transdifferentiation of pancreatic alpha cells to beta cells. We previously reported two small molecules, BRD7389 and GW8510, that induce insulin expression in a mouse alpha cell line and provide a glimpse into potential intermediate cell states in beta-cell reprogramming from alpha cells. These small-molecule studies suggested that inhibition of kinases in particular may induce the expression of several beta-cell markers in alpha cells. To identify potential lineage reprogramming protein targets, we compared the transcriptome, proteome, and phosphoproteome of alpha cells, beta cells, and compound-treated alpha cells. Our phosphoproteomic analysis indicated that two kinases, BRSK1 and CAMKK2, exhibit decreased phosphorylation in beta cells compared to alpha cells, and in compound-treated alpha cells compared to DMSO-treated alpha cells. Knock-down of these kinases in alpha cells resulted in expression of key beta-cell markers. These results provide evidence that perturbation of the kinome may be important for lineage reprogramming of alpha cells to beta cells.

## Introduction

Type-1 diabetes (T1D) is a chronic autoimmune disease affecting 35 million patients worldwide. In T1D, insulin-secreting pancreatic beta cells are destroyed by autoreactive immune cells [1], [2]._ENREF_1 The most common treatment for T1D is daily injection of insulin; however, this treatment cannot always ensure optimal glucose homeostasis, leading to complications such as blindness, heart disease, limb amputation, and ultimately death [3]. Another therapeutic strategy involves transplantation of pancreatic islets [4] via infusion through the portal vein into the liver, but high cost [5], limited donor availability, and beta-cell toxicity [6], [7] of immunosuppressive drugs severely restrict the use of this treatment protocol.

Since T1D is characterized by extreme loss of beta-cell mass, replenishing the beta-cell population by converting other pancreatic cell types, such as alpha cells, to beta cells may be a viable therapeutic strategy [8], [9]. For example, lineage reprogramming of pancreatic alpha cells to beta cells by ectopic expression of transcription factor, *Pax4*, has been shown to restore normoglycemia in mouse models of diabetes [10]. An alternative, and potentially more therapeutically promising, approach for lineage reprogramming involves the use of small molecules. This approach, which we term chemical transdifferentiation [11], is potentially safer, as it does not require viral delivery, and offers greater dosage and temporal control. However, identification of proteins or pathways that can be targeted for chemical transdifferentiation requires knowledge of the similarities and differences in the molecular and physiological architecture of the initial state and the final, transdifferentiated state.

To gain such knowledge, we compared the transcriptomes and proteomes of alpha and beta cell lines. In addition to analyzing the basal state in cell culture, we also used two small molecules, BRD7389 and GW8510, that we previously discovered to induce insulin expression in alpha cells (Figure S1 in File S1) [12], [13]. BRD7389 and GW8510 inhibit several kinases, such as the RSK family and CDK2, suggesting that lineage reprogramming of alpha cells to insulin-producing cells may involve downregulation of specific phosphorylated proteins in alpha cells. We reasoned that a signature of such phosphorylated proteins would be its presence at a higher level in alpha cells compared to beta cells; further, BRD7389 and GW8510 treatment of alpha cells should lower the levels of such phosphorylated proteins, as the alpha cells progress towards an intermediary cell state. To identify phosphoproteins with these signatures, we compared the phosphoproteomes of alpha and beta cell lines, and also examined the alterations in the phosphoproteomes of alpha cell line upon treatment with either BRD7389 or GW8510.

We identified two proteins, BR serine/threonine kinase 1 (Brsk1) and calcium/calmodulin-dependent protein kinase kinase 2 (Camkk2), which were each phosphorylated at higher levels for specific phosphosites in alpha cells as compared to those in beta cells. Moreover, both BRD7389 and GW8510 treatment of alpha cells led to decreased phosphorylation at these phosphosites for both proteins. Knock-down of either kinase was sufficient to induce mRNA and protein expression of beta-cell markers, including insulin, in alpha cells. Furthermore, inhibitors of Camkk2 pathway induced beta-cell markers in alpha cells. These studies highlight the importance of fully characterizing cell states using both genetic and proteomic technologies in developing a cellular reprogramming strategy.

## Materials and Methods

### Cell culture and reagents

Mouse pancreatic cell lines αTC1 and βTC3 were obtained from ATCC and cultured at 37°C and 5% CO_2_ in low-glucose (1 g/L) DMEM supplemented with 10% fetal bovine serum (Hyclone), 50 U/mL penicillin and 50 µg/mL streptomycin. Media was changed every 3–4 days. KN62 and KN93 were obtained from Sigma-Aldrich.

### Microarray analyses

GenePattern was used to perform Gene Set Enrichment Analysis (GSEA) on previously reported data [14], using the gene set database c2.all.v3.0.symbols.gmt, chip platform mouse 430a_2.chip, and 1000 permutations. GENE-E [15] was used for comparative marker selection, and to generate the heat map. The data are available as GEO accession number GSE36379.

### Cell mitochondrial respiration and glycolytic activity

αTC1 cells were treated with 0.1% DMSO or 0.85 µM BRD7389 for 3 days. Cells were seeded at 40,000/well into polyornithine pre-coated 96-well cell culture plates from Seahorse Bioscience. Seahorse Bioscience XF-96 extracellular flux analyzer was used to measure OCR and ECAR over time and analyte addition. Cell glycolytic activity was assessed by adding 10 mM glucose at 34 min, while the maximum glycolytic activity was induced with 5 µM oligomycin at 55 min. Glycolytic reserve was determined by adding the glucose analogue 2-deoxyglucose (100 mM) into the cell media at 76 min. Mitochondrial respiration was assessed by adding: 1 µM oligomycin into the media at 34 min, 1 µM of the ionophore CCCP, and a combination of 5 µM rotenone and 5 µM antimycin A at 76 min. αTC1 cells media contained 0.1% DMSO.

### SILAC experiments

αTC1 and βTC3 and cells were cultured in low-glucose (1 g/L) DMEM media (custom preparation from Caisson Laboratories) that was deficient in L-arginine and L-lysine. DMEM media was supplemented with 10% dialyzed FBS (Sigma-Aldrich), penicillin, streptomycin, glutamine, and either L-arginine (Arg 0) and L-lysine (Lys 0), L-arginine ^13^C_6_-HCL (Arg 6) and L-lysine-4,4,5,5d_4_ (Lys 4), or L-arginine ^13^C_6_-^15^N_4_-HCl (Arg 10) and L-lysine ^13^C_6_, ^15^N_2_-HCl (Lys 8) (Sigma-Aldrich). To achieve >95% SILAC amino acid incorporation, cells were grown for 25 days. For alpha and beta cell comparison experiments, αTC1 cells were grown in heavy state (R10K8), βTC3 were grown in light state (R0K0), and the medium state (R6K4) contained a 1∶1 mix of αTC1 and βTC3 cells. These experiments were completed in biological replicates with SILAC label swapping. After compound treatment, cells were washed with 1x PBS.

Cells were lysed at 4°C using a buffer containing 8 M urea, 50 mM Tris-HCl pH 7.5, 150 mM NaCl, 1 mM EDTA, 2 µg/µl aprotinin (Sigma-Aldrich), 10 µg/µl leupeptin (Roche), 1 mM phenylmethylsulfonyl fluoride (PMSF), 10 mM NaF, 2 mM Na_3_VO_4_, 50 ng/ml calyculin A (Calbiochem), phosphatase inhibitor mixture 1 (1/100, Sigma) and phosphatase inhibitor mixture 2 (1/100, Sigma). Cellular lysates were spun down at 20,000×g at 4°C to remove insoluble material. Protein concentrations were determined using a bicinchoninic acid (BCA) protein assay (Pierce) and corresponding SILAC samples were combined in equal ratio. Approximately 5 mg of protein was used per SILAC state. Proteins were reduced with 5 mM dithiothreitol and subsequently alkylated with 10 mM iodoacetamide. Prior to digestion, samples were diluted 1∶4 with 50 mM Tris-HCl pH 7.5. Proteins were digested with sequencing grade modified trypsin (Promega) using an enzyme-to-substrate ratio of 1∶50 overnight at 25°C. Samples were acidified with 0.5% formic acid and subsequently desalted using a 500 mg tC18 Sep-Pak SPE cartridge (Waters) as previously described [16].

Non-phosphorylated and phosphorylated peptides were prepared as previously described [16]. Briefly, peptides were fractionated offline by strong cation exchange (SCX) chromatography on an Akta Purifier 10 system (GE Healthcare) using a polysulfoethyl A strong cation exchange (SCX) column from PolyLC (250×9.4 mm, 5 µm particle size, 200 A pore size). A 160 min gradient was utilized for the fractionation with using 7 mM KH_2_PO_4_, pH 2.65, 30% MeCN as solvent A and 7 mM KH_2_PO_4_, pH 2.65, 350 mM KCl, 30% MeCN as Solvent B. For proteome analyses 5% of each SCX fraction was aliquoted, and fractions were combined into 24 proteome samples that were subsequently desalted using StageTips.[17] For phosphoproteome analysis, 95% of each SCX fraction was used, and the SCX fractions were combined into 12 phosphopeptide samples. All SCX fractions were subsequently desalted with reversed phase tC18 SepPak columns.

Phosphopeptide enrichment was completed using immobilized metal affinity chromatography (IMAC) as previously described [16]. Briefly, peptides were reconstituted in 200 µl of 40% MeCN, 0.1% formic acid and incubated for with 10 µl of packed Phos-Select beads (Sigma) for 1 hr. Subsequently, IMAC beads were loaded on C18 StageTips, washed twice with 40% MeCN, 0.1% formic acid and once with 50 µl 1% formic acid. Phosphorylated peptides were then transeluted to the C18 portion of the StageTip by washing with 70 µl of 500 mM K_2_HPO_4_ (pH 7.0) three times. StageTips were then washed with 50 µl of 1% formic acid and peptides were eluted with 80 µl of 50% MeCN/0.1% formic acid and dried to completeness.

Peptide samples were reconstituted in 3% MeCN/1% formic acid analyzed by LC-MS/MS using an Agilent 1200 LC coupled online to an LTQ Orbitrap Velos mass spectrometer (Thermo Fisher Scientific). Peptides were loaded onto a fused-silica capillary column (New Objective) packed in-house with 14 cm of C18 reversed phase media (3 µm ReproSil-Pur C18-AQ media, Dr. Maisch GmbH) and eluted into the mass spectrometer using a 70 min linear gradient (∼0.29%B/min) from 10% solvent A (0.1% formic acid) to 30% solvent B (0.1% formic acid/90% acetonitrile). The mass spectrometer was operated in the data dependent mode where an MS1 scan (R = 60 K) was acquired with the Orbitrap analyzer followed by acquisition of CID MS/MS scans with the iontrap analyzer on the top 16 most abundant ions. An MS1 ion target of 1×10^6^ ions and an MS2 target of 1×10^4^ ions were utilized for acquisition. The maximum ion time was set to 1 s for MS1 scans and set to 100 ms for MS/MS scans. The dynamic exclusion time was 120 s, the repeat count was set to 2, and the repeat duration was set to 20 s. Monoisotopic precursor selection and non-peptide monoisotopic recognition functions were enabled.

All proteome and phosphoproteome data were processed together using the MaxQuant software package v1.0.13.13 and searched against the International Protein Index protein sequence database (IPI version 3.70, mouse). For searching, the enzyme specificity was set to trypsin, the number of missed cleavages was set to 2, and the MS/MS tolerance was set to 0.5 Da. Oxidation of methionines, N-terminal protein acetylation, and phosphorylation of serines, threonines, and tyrosines were searched as variable modifications, while carbamidomethylation of cysteines was searched as a fixed modification. Peptides harboring oxidized methionines or N-terminal acetylations were used for protein quantification. Log_2_ SILAC ratios of βTC3 versus αTC1 cells followed a normal distribution that was fitted using least-squares regression. Mean and standard deviation values derived from the gaussian fit were used to calculate p-values.

The peptide and protein false discovery rate (FDR) was set to 0.01 and the site FDR was set to 1. Phosphosite localization was completed as described in Mertins *et al*. [18].

### siRNA experiments

Mouse *Brsk1* and *Camkk2* Gene Solution siRNAs (Qiagen) were used to perform the gene knock-down experiments. Quantitect primer sets for each gene were used to determine gene expression. Mouse αTC1 cells were plated in 96-well Corning (3340) plates at 40,000 cells/well in 100 µL DMEM. 0.3 µL/well LipofectamineTM RNAiMAX (Invitrogen) and Opti-MEM Media was used to transfect the mix of 4 different siRNA constructs into cells. Cells were incubated in transfection mix for 6 h at 37°C, before changing to fresh media. Cells were cultured for 3 days, followed by qPCR or immunohistochemistry. Statistical significance was determined using t-test.

### shRNA experiments

The different shRNA lentiviruses were obtained from the RNAi Consortium (TRC; http://www.broadinstitute.org/rnai/public/) in 96-well format, with approximately 108 viral particles/mL per well. Hairpin identities are listed in Table S1 in File S1. Mouse αTC1 cells were plated in 96-well Corning (3340) plates at 40,000 cells/well in 100 µL standard culture media. The next day, polybrene was added to each well (6 µg/mL), and cells were spin-infected with 1 µL virus at 2,250 rpm for 30 min at 30°C. Media was changed 4 h later to fresh media. The next day, media containing 1 µg/mL puromycin was added. Puromycin-supplemented media was changed every 3 days. After 10 days in culture, cells were lysed and mRNA extracted using Qiagen RNeasy 96 Kit. Statistical significance was determined using t-test.

### Immunocytochemistry

Cells were fixed with 4% PFA for 15 min, followed by a 0.2% Triton-X-100 permeabilization for 20 min, and blocking with PBS supplemented with 2% BSA for 2 h. Fixed cells were then incubated with a mix of 1∶200 rabbit anti-Pdx1 (Abcam) and 1∶500 guinea pig anti-insulin (Sigma) overnight at 4°C. As secondary antibodies, AlexaFluor594 anti-rabbit and AlexaFluor488 (Invitrogen) anti-guinea pig were used. Images were acquired on an ImageXpress Micro automated microscope (Molecular Devices). Exposure settings: 600 ms for Pdx1 (TxRed), 200 ms for insulin (FITC), and 8 ms for DAPI. Statistical significance was determined using t-test.

### Gene expression

Total mRNA from siRNA and compound-treated cells were extracted with Qiagen RNeasy Plus Mini Kit. qPCRs were performed with Power SybrGreen PCR Master Mix and an Applied Biosystems 7900HT plate reader. Mouse primers were obtained from IDT and are listed in Table S2 in File S1.

## Results

### Experimental strategy and rationale

**Figure 1:**
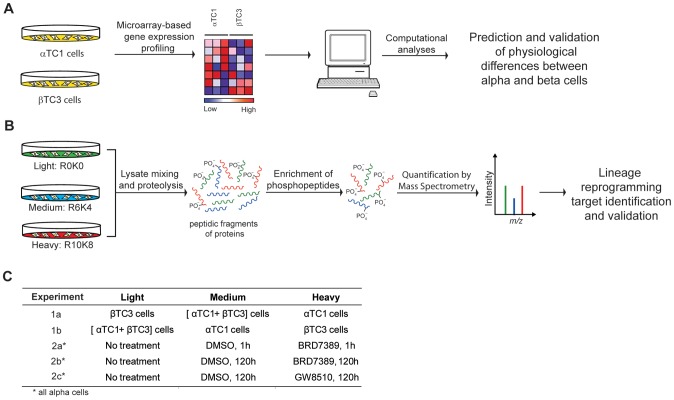
Experimental design for comparative analyses of pancreatic alpha and beta cells using chemical probes, gene-expression analysis, and mass spectrometry-based quantitative phosphoproteomics. (A) Computational prediction and experimental validation of phenotypic differences between mouse alpha cells (αTC1) and beta cells (βTC3) using comparative gene-expression analysis. (B) Workflow for quantitative proteomic and phosphoproteomic analysis of alpha and beta cells, and alpha cells treated with BRD7389 and GW8510. (C) Outline of SILAC conditions upon which quantification was performed. These analyses generated a candidate list of kinases differentially expressed in alpha and beta cells from which target kinases were identified using knockdown, chemical genetics, and immunocytochemistry.

We were interested in comparing the genetic and proteomic levels of various transcription factors in alpha and beta cells, as such proteins are linked with lineage reprogramming. Thus, we compared the gene-expression profiles of the mouse alpha-cell (αTC1) and beta-cell (βTC3) lines (Figure 1A). In parallel, we compared the proteomes and phosphoproteomes of alpha cells and beta cells using stable isotope labeling by amino acids in cell culture (SILAC; Figure 1B) [19]–[21]. We used a three-state labeling strategy, with two states corresponding to the individual cell populations, and the third state corresponding to a mixed proteome from alpha and beta cells. This approach enabled four protein intensity measurements for alpha cells and beta cells using only two replicates (Figure S2 and S3 in File S1). We analyzed the phosphoproteomes of alpha cells and beta cells by enrichment of phosphopeptides using immobilized metal-affinity chromatography (Figure 1B), and also determined the changes in the phosphoproteome of alpha cells upon treatment with BRD7389 (1 h, 120 h) or GW8510 (120 h) (Figure 1C).

### Transcriptome analysis and phenotypic studies

**Figure 2:**
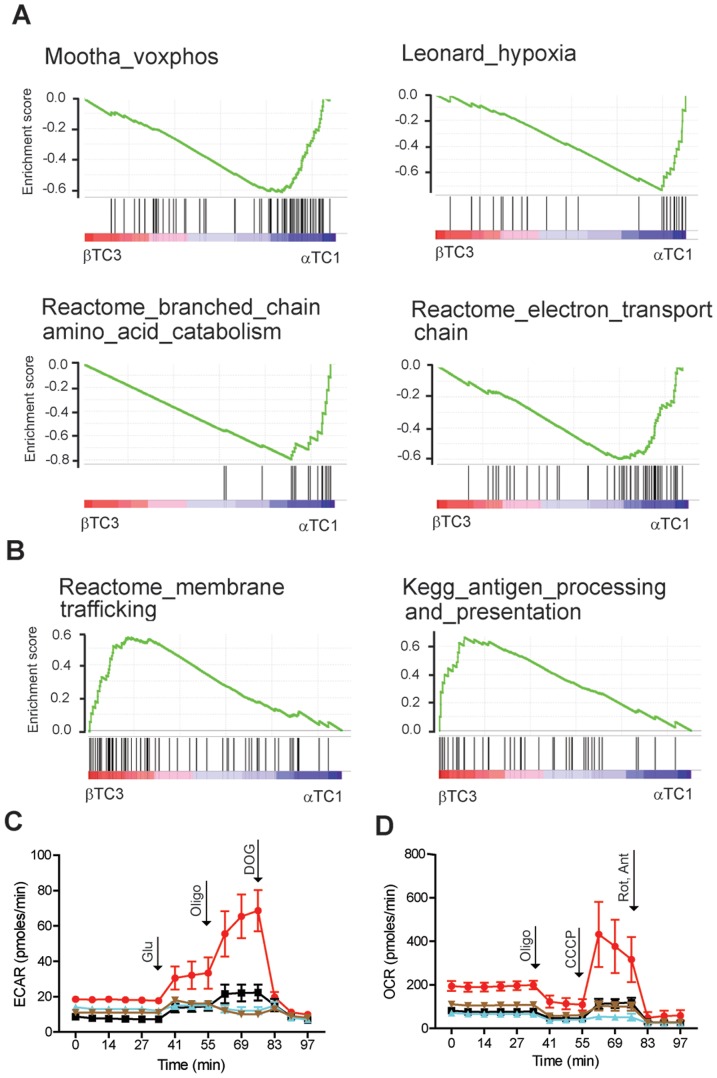
Gene-expression analysis of alpha and beta cell lines reveals higher metabolic activity in the alpha cell line. Gene sets with increased expression in (A) alpha or (B) beta cell lines were identified by performing gene-set enrichment analysis (GSEA) on gene-expression profiling data, resulting in an enrichment score profile for each gene set (green line). Individual members of each gene set (vertical black bars) are enriched in either alpha cells (blue) or beta cells (red). To validate the predicted differences in cellular respiration between alpha and beta cells, we determined (C) extracellular acidification rate (ECAR) and (D) oxygen consumption rate (OCR) of alpha cells (red), BRD7389-treated alpha cells (black), βTC3 cells (blue), and INS-1E cells (brown). Glucose (Glu), oligomycin (Oligo), 2-deoxyglucose (DOG), CCCP, and rotenone/antimycin A (Rot/Ant) were added at the indicated times. Data represent the average and standard deviation of 18 biological replicates.

We used previously described gene-expression profiling data [14], where the transcript levels of ∼14,000 genes of alpha and beta cells were determined using GeneChip Mouse Genome 430A arrays (Affymetrix). A similar comparison of alpha and beta cell transcriptomes has been reported, using cell sorting of primary human islets [22]. To identify the genes most differentially expressed between alpha cells and beta cells, we used comparative marker selection [23], which employs statistical approaches such as false-discovery rate (FDR) and family-wise error rate (FWER) to account for multiple-hypothesis testing. From these top genes, we identified the transcription factors that were differentially expressed in alpha cells and beta cells (Figure S4 in File S1). A number of transcription factors known to be involved in alpha- and beta-cell specification were identified, including *Pdx1*, *Mnx1*, and *Irx1*. To examine the pathways involved with differentially expressed genes, we used gene-set enrichment analysis (GSEA) [24], [25], which determines whether a pre-defined set of genes shows statistically significant, concordant differences between two biological states (Figure 2A, B). We found that, among other gene sets, genes corresponding to oxidative phosphorylation, hypoxia, branched-chain amino acid catabolism, and electron transport were enriched in alpha cells (Figure 2A, Table S3 in File S1). In beta cells, gene sets corresponding to membrane trafficking and antigen presentation and processing were enriched (Figure 2B, Table S4 in File S1).

Since GSEA suggested that the alpha cell line may have a higher metabolic rate than beta cells, we compared the mitochondrial respiration and glycolytic activity in αTC1 cells, BRD7389-treated αTC1 cells, and two beta cell lines: βTC3 and INS-1E cells. We observed higher glycolysis rate, maximum glycolytic capacity, and glycolytic reserve in αTC1 cells, as measured by extracellular acidification rate (Figure 2C, S5 in File S1). We also determined mitochondrial respiration in cell populations by quantifying extracellular oxygen consumption rates (OCR) in the presence of various mitochondrial inhibitors [26]. αTC1 cells had elevated basal respiration rates, energy coupling efficiency, and maximum respiration rate compared to the beta cell lines (Figure 2D). Further, treatment of αTC1 cells with BRD7389 led to a decrease in the overall glycolytic activity and mitochondrial respiration rates, a phenotype reminiscent of the beta cell lines (Figure 2D) [27]–[30], further supporting the hypothesis that BRD7389 directs alpha cells towards an intermediate cell state.

### Proteome and PTM coverage and analysis

We performed quantitative proteomics on the alpha cells, compound-treated alpha cells, and beta cells using SILAC [19]–[21]. Briefly, cells were cultured in media with amino acids containing light, medium, or heavy isotopes of arginine and lysine. Cell lysates from these three SILAC states were mixed and proteins were reduced, alkylated, and digested with trypsin. The SILAC media (*e.g*., light, medium, or heavy) from which each observed tryptic peptide originates determines the isotopic label state of the lysine or arginine residue in each peptide. The peptides were subsequently fractionated using strong cation exchange chromatography and phosphopeptides were enriched using immobilized metal-ion chromatography, where phosphopeptides chelate to a bead-immobilized iron (III) complex. Protein ratios were determined from the Protein Groups table generated by the MaxQuant [31], [32] software package v1.0.13.13. Two independent SILAC triple-labeling experiments were completed for both the proteome and phosphoproteome analyses of alpha cells and beta cells, with Pearson correlation coefficient r = 0.9 between replicates (Figure S3 in File S1). In total, 3,250 proteins and 9,978 phosphopeptides were quantified across both biological replicates. Approximately 10% of proteins had greater than a three-fold difference in abundance between alpha and beta cells (Table 1), including known hormones (e.g., insulin, glucagon) and kinases (Table 2). Exploration of role of these proteins may provide further insight into the similarities and differences between alpha and beta cells. Neuropeptide Y and somatostatin, which are known to inhibit insulin secretion, were expressed at higher levels in beta cells compared to that in alpha cells [33]–[35]. Pdx1 and amylin, which play a key role in several beta-cell functions, were also expressed at higher levels in beta cells. A comparison of the phosphoproteomes of alpha and beta cells revealed interesting differences. For example, while glucagon is expressed at a higher level in alpha cells, the phosphorylation levels at serines 150 and 152 are higher in beta cells. Further, while insulin is expressed at higher levels in beta cells, the level of phosphorylation at threonine 51 is higher in alpha cells. We were able to detect known p38 MAP kinase-mediated phosphorylation of transcription factors MafA and Pdx1 at serine342 and serine 269, respectively [36]–[38].

**Table 1:** Summary of proteomic studies.

	No. of proteins quantified	No. of proteins ratios with *p*<0.01 and >3-fold change
**Total proteome**	3250	306
	No. of peptides quantified	No. of proteins ratios with *p*<0.01 and >6-fold change
**pSTY peptides**	9979*	1056

*including 467 phosphorylated kinase peptides.

Total proteome results based on 24 SCX fractions over two SILAC experiments. pSTY peptide results are based on 12 SCX fractions over two SILAC experiments.

**Table 2:** Proteins with different expression levels in alpha and beta cells.

Leading Protein ID	Gene	Protein Names	β/α (Log_2_)	p-value
IPI00228830	Ube3c	Ubiquitin-protein ligase E3C	2.0634	0.0051
IPI00420577	Cand2	Cullin-associated NEDD8-dissociated protein 2;Cullin-associated and neddylation-dissociated protein 2;p120 CAND2;TBP-interacting protein TIP120B;TBP-interacting protein of 120 kDa B	2.7923	0.0003
IPI00124993	Epha7	Ephrin type-A receptor 7;Tyrosine-protein kinase receptor EHK-3	2.0891	0.0047
IPI00467350	Pbk	Lymphokine-activated killer T-cell-originated protein kinase	2.286	0.0022
IPI00136625	Nagk	N-acetyl-D-glucosamine kinase;GlcNAc kinase	2.3244	0.0019
IPI00222731	Baiap2	Brain-specific angiogenesis inhibitor 1-associated protein 2;Insulin receptor tyrosine kinase 53 kDa substrate	2.5636	0.0007
IPI00119575	Prkar1a	Putative uncharacterized protein;cAMP-dependent protein kinase type I-alpha regulatory subunit	4.081	0.0000
IPI00649296	Camk2b	Calcium/calmodulin-dependent protein kinase II	4.3953	0.0000
IPI00465657	Map3k15	Mitogen-activated protein kinase kinase kinase 15;MAPK/ERK kinase kinase 15	−3.3046	0.0000
IPI00471226	Abt1	Activator of basal transcription 1	−1.9123	0.0097
IPI00928012	Ptprn2	Protein tyrosine phosphatase, receptor type, N polypeptide 2;Receptor-type tyrosine-protein phosphatase N2;PTP IA-2beta;Protein tyrosine phosphatase-NP	2.3397	0.0018
IPI00752710	Cebpz	CCAAT/enhancer-binding protein zeta;CCAAT-box-binding transcription factor	−2.0338	0.0064
IPI00674690	Tcf25	Transcription factor 25;Nuclear localized protein 1;Nuclear localized protein-1 isoform d	2.3231	0.0019
IPI00923679	Irs2	Insulin receptor substrate 2;4PS	−2.1428	0.0044
IPI00222731	Baiap2	Brain-specific angiogenesis inhibitor 1-associated protein 2;Insulin receptor tyrosine kinase 53 kDa substrate;Insulin receptor substrate p53;Insulin receptor substrate protein of 53 kDa;Brain-specific angiogenesis inhibitor 1-associated protein 2, isoform CRA_c	2.5636	0.0007
IPI00132557	Pdx1	Pancreas/duodenum homeobox protein 1;Insulin promoter factor 1;Islet/duodenum homeobox 1;Somatostatin-transactivating factor 1	2.6903	0.0004
IPI00471240	Insm1	Insulinoma-associated protein 1;Zinc finger protein IA-1	3.9295	0.0000
IPI00134310	Ins1	Insulin-1;Insulin-1 B chain;Insulin-1 A chain	4.4525	0.0000
IPI00134311	Ins2	Insulin-2;Insulin-2 B chain;Insulin-2 A chain	5.1812	0.0000
IPI00886028	Igf2	Insulin-like growth factor II;Multiplication-stimulating polypeptide;IGF-II;Preptin	6.1304	0.0000
IPI00135645	Gcg	Glucagon	−4.2082	0.0000
IPI00121190	Egfr	Epidermal growth factor receptor	−2.0428	0.0062
IPI00230133	Hist1h1b	Histone H1.5;H1 VAR.5;H1b	−2.0679	0.0057
IPI00223713	Hist1h1c	Histone H1.2;H1 VAR.1;H1c	−2.2984	0.0024
IPI00223714	Hist1h1e	Histone H1.4;H1 VAR.2;H1e	−2.4348	0.0014
IPI00228616	Hist1h1a	Histone H1.1;H1 VAR.3	−2.4669	0.0013
IPI00114333	Rps6ka3	Ribosomal protein S6 kinase alpha-3	1.8178	0.0117

The phosphoproteome of alpha cells was not significantly perturbed in the first hour of BRD7389 treatment, as only 2% of phosphopeptides showed alteration in levels. However, after five days of BRD7389 treatment, 9% of phosphopeptides were upregulated and 5% were downregulated. BRD7389 and GW8510 inhibit RSK and CDK2 kinases, respectively. Hence, we expected a decrease in phosphorylation of protein substrates of Rsk and Cdk2 upon BRD7389 or GW8510 treatment of alpha cells. Accordingly, we observed a decrease in S6 and EF2 phosphorylation, known substrates of RSK and CDK2, respectively (Table S5 in File S1).

### Suppression of Brsk1 and Camkk2 elicits beta cell features in αTC1 cells

**Figure 3:**
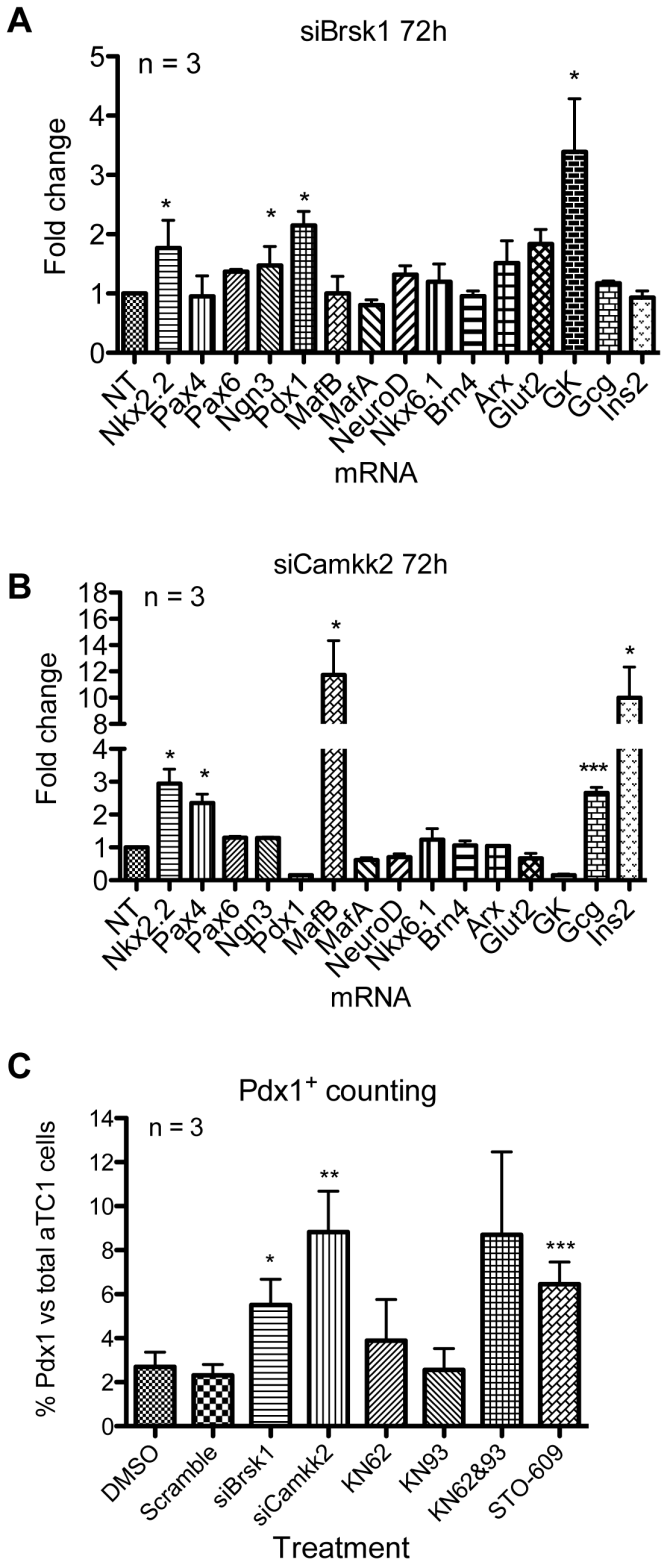
Knock-down of *Brsk1* and *Camkk2* elicit the expression of key beta cell-specific genes in αTC1 cells. mRNA expression of beta and alpha cell-specific genes was measured using qPCR after knock-down of (A) *Brsk1* or (B) *Camkk2*. Fold changes were calculated compared to untransfected control αTC1 cells, and normalized to *Gapdh* expression. Significance was determined by t-test. *p<0.05, ***p<0.005. (C) αTC1 cells transfected with the indicated siRNA, or treated with the indicated compounds, were analyzed for Pdx1 protein expression by immunofluorescence. The percentage of Pdx1^+^ cells was calculated for each treatment. The significance was determined by t-test. *p<0.05, **p<0.01, and ***p<0.001.

Although several kinases were differentially expressed in alpha and beta cells, we focused our attention on BRSK1 and CAMKK2, as they were more highly phosphorylated in alpha cells compared to beta cells, and in untreated alpha cells compared to compound-treated cells (Table S6 in File S1). We were unable to quantify absolute protein levels of these kinases in our proteomic analyses, but gene expression analysis suggested higher mRNA levels of Camkk2 in beta cells. Since the role of these proteins in maintenance of alpha cell state was unknown, we initiated our investigation by performing siRNA-mediated knock-down of *Brsk1* or *Camkk2* in αTC1 cells, and determined the expression of alpha and beta cell-specific genes. *Brsk1* and *Camkk2* mRNA levels were reduced by 64% and 85%, respectively, after three-day siRNA transfection (Figure S6 in File S1). Expression of *Camkk2* was also reduced in si*Brsk1*-treated cells (53%), suggesting that Brsk1 may be an upstream activator of Camkk2. *Brsk1* knock-down resulted in the upregulation of the mRNA levels of genes important to beta-cell identity, including *Pdx1*, *Ngn3*, *Nkx2.2*, and glucokinase (*Gck*) (Figure 3A), while *Camkk2* down-regulation elicited the expression of *Nkx2.2*, *Pax4*, *MafB*, and the hormones glucagon (*Gcg*) and insulin (*Ins2*) (Figure 3B). Modest levels of Pdx1 protein expression were detected by immunofluorescence in si*Brsk1*- and si*Camkk2*-treated cells (Figure S6 in File S1). Consistent with these observations, STO-609, a Camkk antagonist, and KN62 and KN93, two selective Ca^2+^/calmodulin-dependent protein kinase II (CaMKII) inhibitors, also increased Pdx1 protein expression in αTC1 cells (Figure 3C).

**Figure 4:**
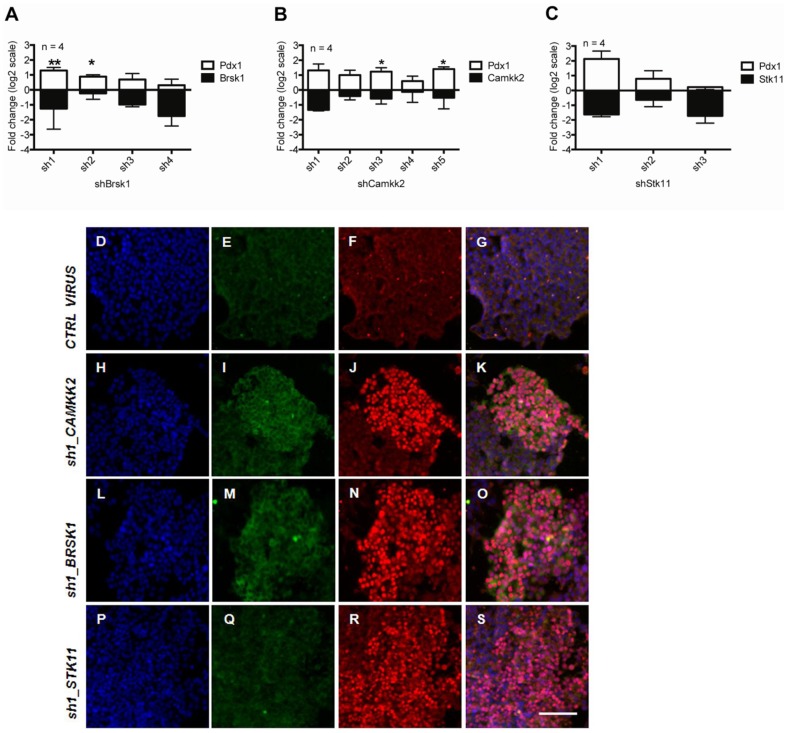
Longer-term lentiviral knockdown of *Brsk1* and *Camkk2* stimulates PDX1 and insulin protein expression in αTC1 cells. Cells were infected with lentivirus carrying expression cassettes that encode shRNAs against (A) *Brsk1*, (B) *Camkk2*, (C) *Stk11*, and were selected with puromycin for 10 days. *Pdx1* mRNA expression (white bars) was measured jointly with the expression of each of the shRNA-targeted genes (black bars). Data represent fold change (in log_2_ scale), compared to the average of control empty vectors. Data represent mean and standard deviation of four independent experiments. The significance was determined by t-test. *p<0.05 and **p<0.01. (D–S) Nuclei (blue), insulin (green), and Pdx1 (red) immunofluorescence, and merged images in 10-day shRNA-expressing αTC1 cells: (D–G) control virus, (H–K) sh1_Camkk2, (L–O) sh1_Brsk1, (P–S) sh1_Stk11. Scale bar = 100 µm.

Since PDX1 protein levels after 3 days of gene knock-down were detectable but faint, we performed a 10-day knock-down of *Brsk1*, *Camkk2*, and *Stk11*, an upstream modulator of *Brsk1*, using lentiviral shRNAs (Figure 4). When using hairpins targeting *Brsk1* and *Camkk2*, a decrease in these kinases corresponded to a concomitant increase in *Pdx1* mRNA expression (Figure 4A–B). Hairpins targeting *Stk11* (two out of three) also increased *Pdx1* expression (Figure 4C). The fact that not all hairpins had the same effect might reflect off-target silencing effects of these hairpins, which are part of the limitations of using small-interfering RNAs [39]. Moreover, individual hairpins to *Camkk2*, *Brsk1*, and *Stk11* elicited the expression of Pdx1 protein, as detected by immunofluorescence (Figure 4D–S). PDX1-positive cells also co-stained for insulin when *Brsk1* or *Camkk2* were knocked down, and more modestly with knock-down of *Stk11* (Figure 4 I,M,Q). Taken together, our results suggest a role for CAMK family proteins in maintaining an alpha-cell phenotype.

## Discussion

In this study, we compared the transcriptomes, proteomes, and phosphoproteomes of a pancreatic alpha and beta cell line, with the aim of identifying new targets whose perturbation may induce expression of beta-cell markers in alpha cells. Gene-expression analysis suggested enhanced mitochondrial respiration and glycolysis in alpha cells compared to beta cells, which we confirmed experimentally. Furthermore, BRD7389 treatment of αTC1 lowered mitochondrial respiration and glycolytic activity as these cells progressed towards a state intermediate between alpha and beta cells. Beta cell lines, in particular INS-1E, have been reported to have high proton leak through the mitochondrial membrane [27], and to have strongly reduced lactate production [28]–[30], which is consistent with our observed low spare respiration and glycolytic capacity. The observed differences in the mitochondrial respiration and glycolytic activity could also arise from differences in the growth rate of the alpha and beta cell lines. Indeed, the beta cell lines used herein have a lower growth rate (doubling time: 2–3 days) compared to that of αTC1 cells (doubling time: 1–2 days).

Most lineage-reprogramming efforts involve overexpression of transcription factors that occur at higher levels in the target cell type. We have identified several transcription factors that are enriched in alpha cells and beta cells in our microarray data. Expected genes were readily identified; for example, Pdx1 plays an important role in beta cell maturation and function [40]. Interestingly, one of the identified transcription factors, motor neuron and pancreas homeobox 1 (Mnx1), was recently reported to play an important role in cell fate determination in zebrafish [41]. Physiological differences between alpha and beta cells are also reflected in the annotated gene sets enriched in these cells. Genes involved in respiration and branched-chain amino acid were enriched in alpha cells, while the genes associated with membrane trafficking and lysosomes were enriched in beta cells, pointing to the relative importance of these biological processes in the two cell types.

Our prior studies suggested that the inhibition of specific kinases in alpha cells can lead to expression of key markers of beta cells [12], [13]. Here, we identified two kinases whose knock-down induced beta-cell markers in alpha cells. BRSK1, also known as SAD-B, is a serine/threonine kinase strongly expressed in mammalian forebrain to determine neuronal polarization [42]. BRSK1 is activated both *in vitro* and *in vivo* by serine/threonine-protein kinase 11 (STK11), also known as liver kinase B1 (LKB1), which plays an important role in the polarization of cortical axons [43]–[45]. STK11 is in turn a tumor suppressor and regulator of energy homeostasis through the activation of AMP-activated protein kinase (AMPK) [46]. A beta cell-specific deletion of Stk11 causes an increase in beta cell-mass and insulin secretion, effects mediated by an activation of the mTOR pathway [47]. In combination with STK11, CAMKK2 (Camkkβ) phosphorylates and activates AMPK [48], [49], while in combination with CAMKK1 (Camkkα), it phosphorylates and activates CAMK1 and CAMK4 [50]. *Camkk2* null mice are protected from obesity, insulin resistance, and glucose intolerance [51]. It is noteworthy that downregulation of Camkk2 in alpha cells increases the expression of both insulin and glucagon. Directed differentiation of embryonic stem cells into mature hormone secreting endocrine cells similarly involve polyhormonal cellular states, often described as a hallmark of non-fully differentiated cells. Interestingly, Camkk2 has been reported to play a role in neuronal differentiation[52] and bone remodeling by stimulating osteoblast formation[53] further supporting the role of this kinase to impact cell fate decision.

This chemical-genetic and phosphoproteomic study provides evidence that perturbing the alpha-cell kinome may be an attractive approach for lineage reprogramming, and points to a potential role for BRSK1, the CAMK kinase family, and STK11 in determining endocrine-cell identity. Gene silencing and compound treatments induced mRNA and protein expression of key beta cell-specific genes, such as Pdx1 and insulin; the LKB1/BRSK1 and CAMKK2 pathways might converge at the level of AMPK, as suggested by previous work [46], [51]. The presence of detectable levels of both glucagon and insulin in both αTC1 and βTC3 cells suggests that these cell lines are not perfect models of alpha and beta cells. However, we have previously shown that the protein targets identified from these cell lines using small molecules, BRD7389 and GW8510, translate in native pancreatic cells of humans.[12], [13] These findings support the notion that these cell lines can lead to the discovery of novel protein targets relevant for the primary cells. A potential drawback of our study is the assumption that phosphorylation of kinases is correlated with kinase activation. Many examples exist where kinases are negatively regulated by phosphorylation (e.g., glycogen synthase kinase 3β). Further studies are required to dissect the opposing actions of kinases and reprogramming. This may include delineation of the signaling pathways of these kinases in endocrine cells, and understanding the signaling role of their phosphorylated forms. We anticipate that such studies may spur development of small molecules for lineage reprogramming of alpha cells to insulin-producing cells.

## Supporting Information

File S1 Six supplementary figures (Figures S1–S6) and six supplementary tables (Tables S1–S6).
(DOC)

File S2 Phosphorproteomic data for compound treatments.
(XLSB)

File S3 Phosphoproteomic data comparing αTC and βTC cells.
(XLSX)

File S4 Total proteomic data comparing αTC and βTC cells.
(XLS)

## References

[1] Gepts W (1965) Pathologic anatomy of the pancreas in juvenile diabetes mellitus. Diabetes 14: 619–633. 5318831 10.2337/diab.14.10.619

[2] van Belle TL, Coppieters KT, von Herrath MG (2011) Type 1 diabetes: etiology, immunology, and therapeutic strategies. Physiol Rev 91: 79–118. 21248163 10.1152/physrev.00003.2010

[3] Hirsch IB, Farkas-Hirsch R, Skyler JS (1990) Intensive insulin therapy for treatment of type I diabetes. Diabetes Care 13: 1265–1283. 2276310 10.2337/diacare.13.12.1265

[4] Rajab A (2010) Islet transplantation: alternative sites. Curr Diab Rep 10: 332–337. 20665132 10.1007/s11892-010-0130-6

[5] de Kort H, de Koning EJ, Rabelink TJ, Bruijn JA, Bajema IM (2011) Islet transplantation in type 1 diabetes. BMJ 342: d217. 21257658 10.1136/bmj.d217

[6] Chatenoud L (2008) Chemical immunosuppression in islet transplantation—friend or foe? N Engl J Med 358: 1192–1193. 18337609 10.1056/NEJMcibr0708067

[7] Johnson JD, Ao Z, Ao P, Li H, Dai LJ, et al (2009) Different effects of FK506, rapamycin, and mycophenolate mofetil on glucose-stimulated insulin release and apoptosis in human islets. Cell Transplant 18: 833–845. 19500470 10.3727/096368909X471198

[8] Borowiak M, Melton DA (2009) How to make β cells? Curr Opin Cell Biol 21: 727–732. 19781928 10.1016/j.ceb.2009.08.006 PMC4617625

[9] Yechoor V, Chan L (2010) Minireview: beta-cell replacement therapy for diabetes in the 21st century: manipulation of cell fate by directed differentiation. Mol Endocrinol 24: 1501–1511. 20219891 10.1210/me.2009-0311 PMC2940465

[10] Collombat P, Xu X, Ravassard P, Sosa-Pineda B, Dussaud S, et al (2009) The ectopic expression of Pax4 in the mouse pancreas converts progenitor cells into alpha and subsequently beta cells. Cell 138: 449–462. 19665969 10.1016/j.cell.2009.05.035 PMC2792203

[11] Wagner BK (2010) Grand challenge commentary: Chemical transdifferentiation and regenerative medicine. Nat Chem Biol 6: 877–879. 21079596 10.1038/nchembio.472

[12] Fomina-Yadlin D, Kubicek S, Walpita D, Dancik V, Hecksher-Sorensen J, et al (2010) Small-molecule inducers of insulin expression in pancreatic alpha-cells. Proc Natl Acad Sci U S A 107: 15099–15104. 20696901 10.1073/pnas.1010018107 PMC2930573

[13] Fomina-Yadlin D, Kubicek S, Vetere A, He KH, Schreiber SL, et al (2012) GW8510 Increases Insulin Expression in Pancreatic Alpha Cells through Activation of p53 Transcriptional Activity. PLoS One 7: e28808. 22242153 10.1371/journal.pone.0028808 PMC3252286

[14] Kubicek S, Gilbert JC, Fomina-Yadlin D, Gitlin AD, Yuan Y, et al. (2012) Chromatin-targeting small molecules cause class-specific transcriptional changes in pancreatic endocrine cells. Proc Natl Acad Sci U S A: 5364–5369. 10.1073/pnas.1201079109 PMC3325696 22434908

[15] GENE-E software application:http://www.broadinstitute.org/cancer/software/GENE-E.Accessed 2014 March 27.

[16] Mertins P, Udeshi ND, Clauser KR, Mani DR, Patel J, et al (2012) iTRAQ labeling is superior to mTRAQ for quantitative global proteomics and phosphoproteomics. Molecular & cellular proteomics: MCP 11: M111.014423. 10.1074/mcp.M111.014423 PMC3433912 22210691

[17] Rappsilber J, Mann M, Ishihama Y (2007) Protocol for micro-purification, enrichment, pre-fractionation and storage of peptides for proteomics using StageTips. Nat Protocols 2: 1896–1906. 17703201 10.1038/nprot.2007.261

[18] Mertins P, Udeshi ND, Clauser KR, Mani DR, Patel J, et al. (2012) iTRAQ Labeling is Superior to mTRAQ for Quantitative Global Proteomics and Phosphoproteomics. Mol Cell Proteomics 11. 10.1074/mcp.M111.014423 PMC3433912 22210691

[19] Ong SE, Mann M (2005) Mass spectrometry-based proteomics turns quantitative. Nat Chem Biol 1: 252–262. 16408053 10.1038/nchembio736

[20] Ong SE, Mann M (2006) A practical recipe for stable isotope labeling by amino acids in cell culture (SILAC). Nat Protoc 1: 2650–2660. 17406521 10.1038/nprot.2006.427

[21] Ong SE, Mann M (2007) Stable isotope labeling by amino acids in cell culture for quantitative proteomics. Methods Mol Biol 359: 37–52. 17484109 10.1007/978-1-59745-255-7_3

[22] Dorrell C, Schug J, Lin CF, Canaday PS, Fox AJ, et al (2011) Transcriptomes of the major human pancreatic cell types. Diabetologia 54: 2832–2844. 21882062 10.1007/s00125-011-2283-5 PMC3880150

[23] Reich M, Liefeld T, Gould J, Lerner J, Tamayo P, et al (2006) GenePattern 2.0. Nat Genet 38: 500–501. 16642009 10.1038/ng0506-500

[24] Subramanian A, Tamayo P, Mootha VK, Mukherjee S, Ebert BL, et al (2005) Gene set enrichment analysis: a knowledge-based approach for interpreting genome-wide expression profiles. Proc Natl Acad Sci U S A 102: 15545–15550. 16199517 10.1073/pnas.0506580102 PMC1239896

[25] Mootha VK, Lindgren CM, Eriksson KF, Subramanian A, Sihag S, et al (2003) PGC-1alpha-responsive genes involved in oxidative phosphorylation are coordinately downregulated in human diabetes. Nat Genet 34: 267–273. 12808457 10.1038/ng1180

[26] Brand MD, Nicholls DG (2011) Assessing mitochondrial dysfunction in cells. Biochem J 435: 297–312. 21726199 10.1042/BJ20110162 PMC3076726

[27] Affourtit C, Brand MD (2008) Uncoupling protein-2 contributes significantly to high mitochondrial proton leak in INS-1E insulinoma cells and attenuates glucose-stimulated insulin secretion. Biochem J 409: 199–204. 17824844 10.1042/BJ20070954

[28] Thorrez L, Laudadio I, Deun KV, Quintens R, Hendrickx N, et al (2011) Tissue-specific disallowance of housekeeping genes: The other face of cell differentiation. Genome Res 21: 95–105. 21088282 10.1101/gr.109173.110 PMC3012930

[29] Sekine N, Cirulli V, Regazzi R, Brown LJ, Gine E, et al (1994) Low lactate dehydrogenase and high mitochondrial glycerol phosphate dehydrogenase in pancreatic beta-cells. Potential role in nutrient sensing. J Biol Chem 269: 4895–4902. 8106462

[30] Schuit F, De Vos A, Farfari S, Moens K, Pipeleers D, et al (1997) Metabolic Fate of Glucose in Purified Islet Cells: GLUCOSE-REGULATED ANAPLEROSIS IN β CELLS. J Biol Chem 272: 18572–18579. 9228023 10.1074/jbc.272.30.18572

[31] Cox J, Matic I, Hilger M, Nagaraj N, Selbach M, et al (2009) A practical guide to the MaxQuant computational platform for SILAC-based quantitative proteomics. Nat Prot 4: 698–705. 10.1038/nprot.2009.36 19373234

[32] Cox J, Mann M (2008) MaxQuant enables high peptide identification rates, individualized p.p.b.-range mass accuracies and proteome-wide protein quantification. Nat Biotech 26: 1367–1372. 10.1038/nbt.1511 19029910

[33] Skoglund G, Gross R, Ahrén B, Loubatières-Mariani M-M (1993) Different mechanisms are involved in neuropeptide Y-induced pancreatic vasoconstriction and inhibition of insulin secretion. Eur Jour Pharm 236: 69–74. 10.1016/0014-2999(93)90228-a 8100529

[34] Moltz JH, McDonald JK (1985) Neuropeptide Y: Direct and indirect action on insulin secretion in the rat. Peptides 6: 1155–1159. 3914635 10.1016/0196-9781(85)90443-7

[35] Strowski MZ, Parmar RM, Blake AD, Schaeffer JM (2000) Somatostatin Inhibits Insulin and Glucagon Secretion via Two Receptor Subtypes: An in Vitro Study of Pancreatic Islets from Somatostatin Receptor 2 Knockout Mice. Endocrinology 141: 111–117. 10614629 10.1210/endo.141.1.7263

[36] Zhou G, Wang h, Liu S-H, Shahi KM, Lin X, et al (2013) p38 MAP kinase interacts with and stabilizes pancreatic and duodenal homeobox-1. Curr Mol Med 13: 377–386. 23331010

[37] Guo S, Vanderford NL, Stein R (2010) Phosphorylation within the MafA N Terminus Regulates C-terminal Dimerization and DNA Binding. J Biol Chem 285: 12655–12661. 20208071 10.1074/jbc.M110.105759 PMC2857093

[38] Sii-Felice K, Pouponnot C, Gillet S, Lecoin L, Girault J-A, et al (2005) MafA transcription factor is phosphorylated by p38 MAP kinase. FEBS Lett 579: 3547–3554. 15963504 10.1016/j.febslet.2005.04.086

[39] Bora RS, Gupta D, Mukkur TK, Saini KS (2012) RNA interference therapeutics for cancer: challenges and opportunities (review). Mol Med Report 6: 9–15. 10.3892/mmr.2012.871 22576734

[40] Iype T, Francis J, Garmey JC, Schisler JC, Nesher R, et al (2005) Mechanism of insulin gene regulation by the pancreatic transcription factor Pdx-1: application of pre-mRNA analysis and chromatin immunoprecipitation to assess formation of functional transcriptional complexes. J Biol Chem 280: 16798–16807. 15743769 10.1074/jbc.M414381200

[41] Dalgin G, Ward AB, Hao LT, Beattie CE, Nechiporuk A, et al (2011) Zebrafish mnx1 controls cell fate choice in the developing endocrine pancreas. Development 138: 4597–4608. 21989909 10.1242/dev.067736 PMC3190380

[42] Kishi M, Pan YA, Crump JG, Sanes JR (2005) Mammalian SAD kinases are required for neuronal polarization. Science 307: 929–932. 15705853 10.1126/science.1107403

[43] Barnes AP, Lilley BN, Pan YA, Plummer LJ, Powell AW, et al (2007) LKB1 and SAD kinases define a pathway required for the polarization of cortical neurons. Cell 129: 549–563. 17482548 10.1016/j.cell.2007.03.025

[44] Bright NJ, Carling D, Thornton C (2008) Investigating the regulation of brain-specific kinases 1 and 2 by phosphorylation. J Biol Chem 283: 14946–14954. 18339622 10.1074/jbc.M710381200 PMC3258900

[45] Lizcano JM, Goransson O, Toth R, Deak M, Morrice NA, et al (2004) LKB1 is a master kinase that activates 13 kinases of the AMPK subfamily, including MARK/PAR-1. EMBO J 23: 833–843. 14976552 10.1038/sj.emboj.7600110 PMC381014

[46] Shaw RJ, Lamia KA, Vasquez D, Koo SH, Bardeesy N, et al (2005) The kinase LKB1 mediates glucose homeostasis in liver and therapeutic effects of metformin. Science 310: 1642–1646. 16308421 10.1126/science.1120781 PMC3074427

[47] Fu A, Ng AC, Depatie C, Wijesekara N, He Y, et al (2009) Loss of Lkb1 in adult beta cells increases beta cell mass and enhances glucose tolerance in mice. Cell Metab 10: 285–295. 19808021 10.1016/j.cmet.2009.08.008

[48] Woods A, Dickerson K, Heath R, Hong SP, Momcilovic M, et al (2005) Ca2+/calmodulin-dependent protein kinase kinase-beta acts upstream of AMP-activated protein kinase in mammalian cells. Cell Metab 2: 21–33. 16054096 10.1016/j.cmet.2005.06.005

[49] Hawley SA, Pan DA, Mustard KJ, Ross L, Bain J, et al (2005) Calmodulin-dependent protein kinase kinase-beta is an alternative upstream kinase for AMP-activated protein kinase. Cell Metab 2: 9–19. 16054095 10.1016/j.cmet.2005.05.009

[50] Soderling TR (1999) The Ca-calmodulin-dependent protein kinase cascade. Trends Biochem Sci 24: 232–236. 10366852 10.1016/s0968-0004(99)01383-3

[51] Anderson KA, Ribar TJ, Lin F, Noeldner PK, Green MF, et al (2008) Hypothalamic CaMKK2 contributes to the regulation of energy balance. Cell Metab 7: 377–388. 18460329 10.1016/j.cmet.2008.02.011

[52] Cao W, Sohail M, Liu G, Koumbadinga GA, Lobo VG, et al (2011) Differential effects of PKA-controlled CaMKK2 variants on neuronal differentiation. RNA Biol 8: 1061–1072. 21957496 10.4161/rna.8.6.16691 PMC3256423

[53] Cary RL, Waddell S, Racioppi Li, Long F, Novack DV, et al (2013) Inhibition of Ca2+/Calmodulin–Dependent Protein Kinase Kinase 2 Stimulates Osteoblast Formation and Inhibits Osteoclast Differentiation. J Bone Miner Res 28: 1599–1610. 23408651 10.1002/jbmr.1890 PMC3688641

