# Supplementary material for: Quantitative-Proteomic Comparison of Alpha and Beta Cells to Uncover Novel Targets for Lineage Reprogramming
Source: PLoS One. 2014 Apr 23;9(4):e95194. doi: 10.1371/journal.pone.0095194 (PMC3997365; doi:10.1371/journal.pone.0095194)
Supplement: File S1 — Six supplementary figures (Figures S1–S6) and six supplementary tables (Tables S1–S6). (DOC) [file pone.0095194.s001.doc]

**Quantitative-proteomic comparison of alpha and beta cells to uncover novel targets for lineage reprogramming**

Amit Choudhary^1,2,6^, Kaihui Hu He^2,6^, Philipp Mertins^2^, Namrata D. Udeshi^2^, Vlado Dančík^2^, Dina Fomina-Yadlin^2,3,7^, Stefan Kubicek^2,8^, Paul A. Clemons^2^, Stuart L. Schreiber^2,4,5^, Steven A. Carr^2^, Bridget K. Wagner^2,¶^

^1^Society of Fellows, Harvard University, Cambridge, MA 02138

^2^Broad Institute, Cambridge, MA 02142

^3^Department of Molecular and Cellular Biology, Harvard University, Cambridge, MA 02138

^4^Department of Chemistry and Chemical Biology, Harvard University, Cambridge, MA 02138

^5^Howard Hughes Medical Institute

^6^Equal contributors

^7^Current address: Amgen, 1201 Amgen Court, Seattle, WA 98119

^8^Current address: CeMM Research Center for Molecular Medicine Lazarettgasse 14, AKH BT 25.3, A-1090, Vienna

^¶^To whom correspondence should be addressed: Broad Institute, 7 Cambridge Center, Cambridge, MA 02142. Tel.: 617-714-7363; Fax: 617-714-8969; E-mail: bwagner@broadinstitute.org

| **Page** | **Contents** |
| --- | --- |
| S1 | Table of Contents |
| S2 | Figure S1: Small molecules used in this study |
| S2 | Figure S2: Determination of protein ratios |
| S3 | Figure S3: Proteome and phosphoproteome analysis of βTC3 versus αTC1 cells and comparison of β/α-phosphoproteome differences to BRD7389 or GW8510 perturbation effects in αTC1 cells |
| S4 | Figure S4: Transcription factors enriched in alpha/beta cell |
| S5 | Figure S5: Area under the curve for ECAR and OCR |
| S6 | Figure S6: Gene knockdown of Brsk1 and Camkk2 |
| S7 | Table S1: shRNA hairpins used in this study |
| S8 | Table S2: Primers used in this study |
| S9 | Table S3. Gene sets enriched in alpha cells |
| S10 | Table S4. Gene sets enriched in beta cells |
| S11 | Table S5. Decreased phosphorylation in S6 and EF2 upon treatment of αTC1 cells with BRD7389 or GW8510 treatment |
| S12 | Table S6. Phosphosite ratios of Brsk1 and Camkk2 in αTC1 cells, βTC3cells, and compound-treated αTC1 cells |

**Figure S1.** Small molecules used in this study.


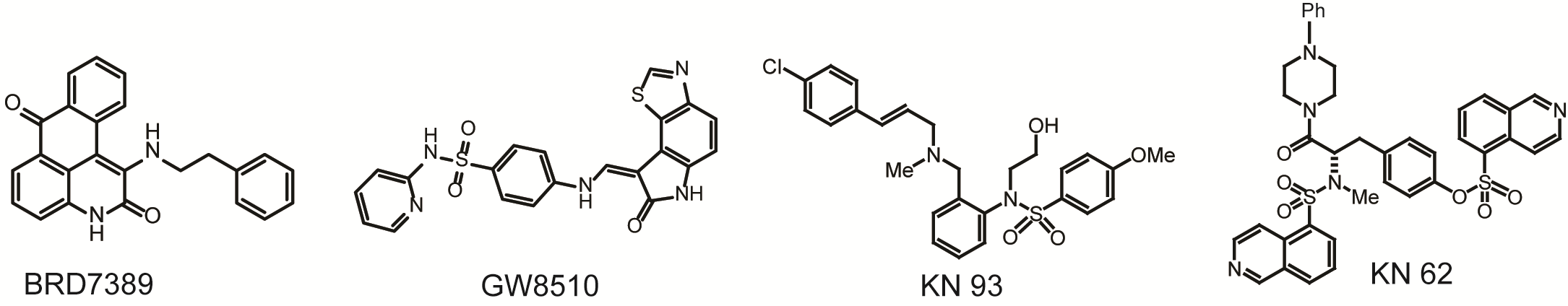


**Figure S2.** Determination of the protein ratios in the SILAC study. The equation used to determine the protein ratios from the mixed proteome is shown below.

**
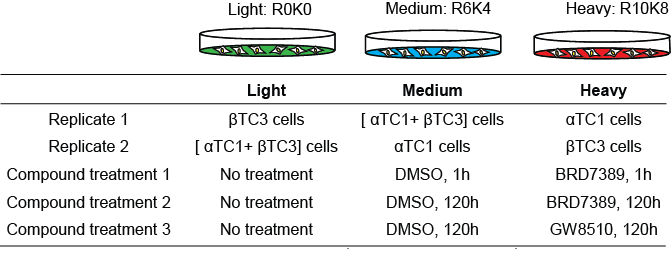
**

- 1

(βTC3 + αTC1)=mix

(βTC3/mix)/(2-( βTC3/mix)) = (βTC3/ αTC1) = (-1) + 2/ (αTC1/mix)

**Figure S3:** Proteome and phosphoproteome analysis of βTC3 versus αTC1 cells and comparison of β/α-phosphoproteome differences to BRD7389 or GW8510 perturbation effects in αTC1 cells. (A) Comparison of βTC3 to αTC1 cells on protein level. Protein SILAC ratios are plotted with frequency distribution histograms in the margins for replicate 1 (Light/Heavy) and replicate 2 (Heavy/Medium). We required 2 or more unique peptides and 3 or more ratio counts per protein and reproducibly quantified 3,241 proteins in both replicates with a Pearson correlation coefficient r = 0.90. (B) Phosphoproteome comparison of βTC3 to αTC1 cells. Phopshopeptide SILAC ratios are plotted with frequency distribution histograms in the margins for replicate 1 (Light/Heavy) and replicate 2 (Heavy/Medium). We reproducibly quantified 4,083 phosphopeptides in both replicates with a Pearson correlation coefficient r = 0.90. (C, D) Comparison of β/α phosphoproteome differences to BRD7389 (C) or GW8510 (D) perturbation effects in αTC1 cells. We quantified an overlap of 3,920 and 3,676 phosphopeptides for the beta/alpha cell experiments and the BRD7389 and GW8510 drug perturbation experiments, respectively. Note the phosphopeptides in the lower left quadrant that are less abundant in βTC3 versus αTC1 cells and also reduced in abundance upon drug treatment with BRD7389 or GW8510. The blue dashed lines indicate 2-fold linear up- or downregulation.


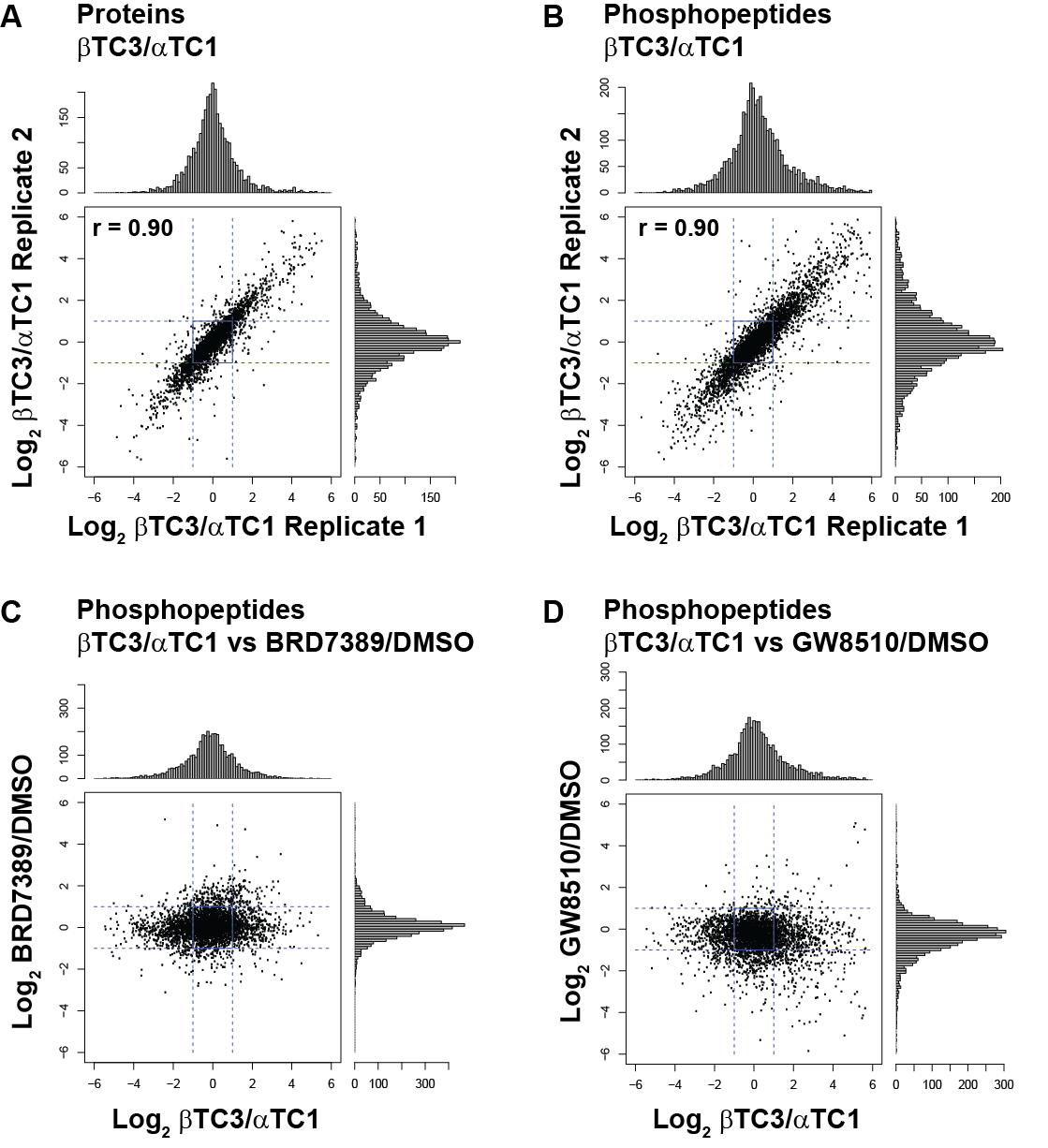


**Figure S4.** Transcription factors enriched in alpha/beta cell. Each row-normalized column represents a biological replicate of each cell type.


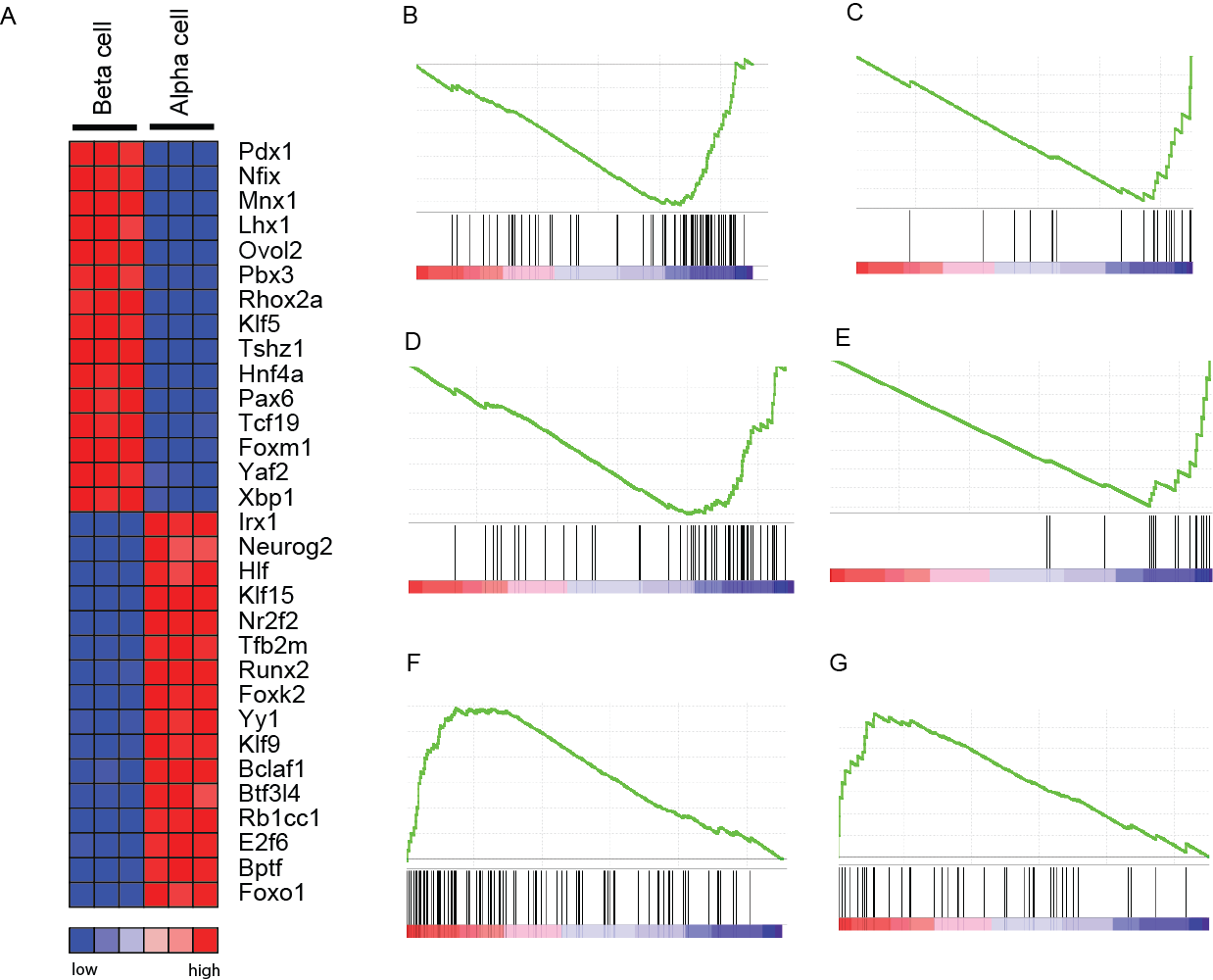


**Figure S5.** Area under the curve for (A) ECAR and (B) OCR. One way Anova followed by Tukey’s multiple comparison tests was performed to determine statistical significance. **p*<0.05, ***p*<0.01, ****p*<0.001, and **** *p*<0.0001.

| **A** |
| --- |
| **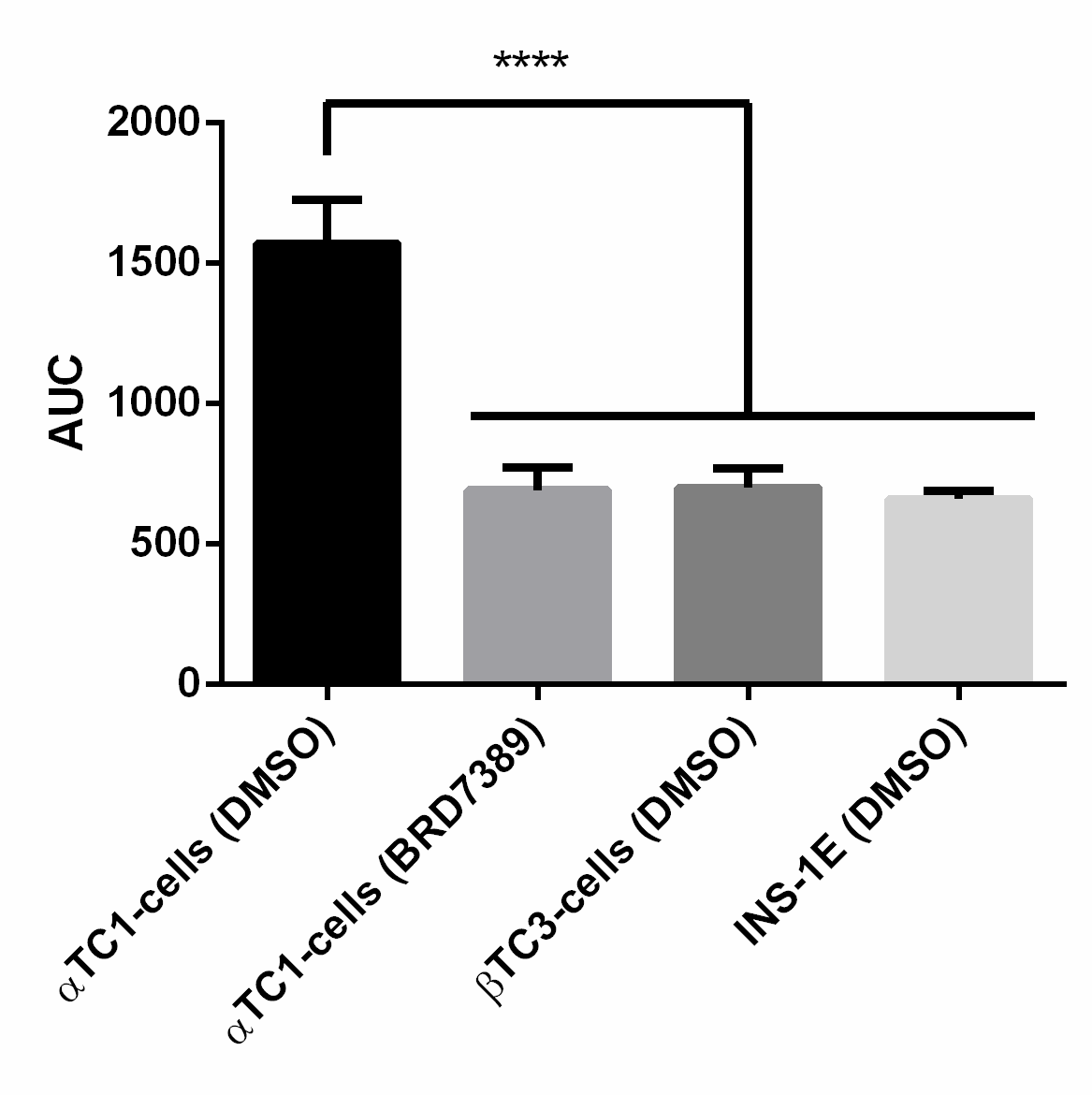** |
| **B** |
| **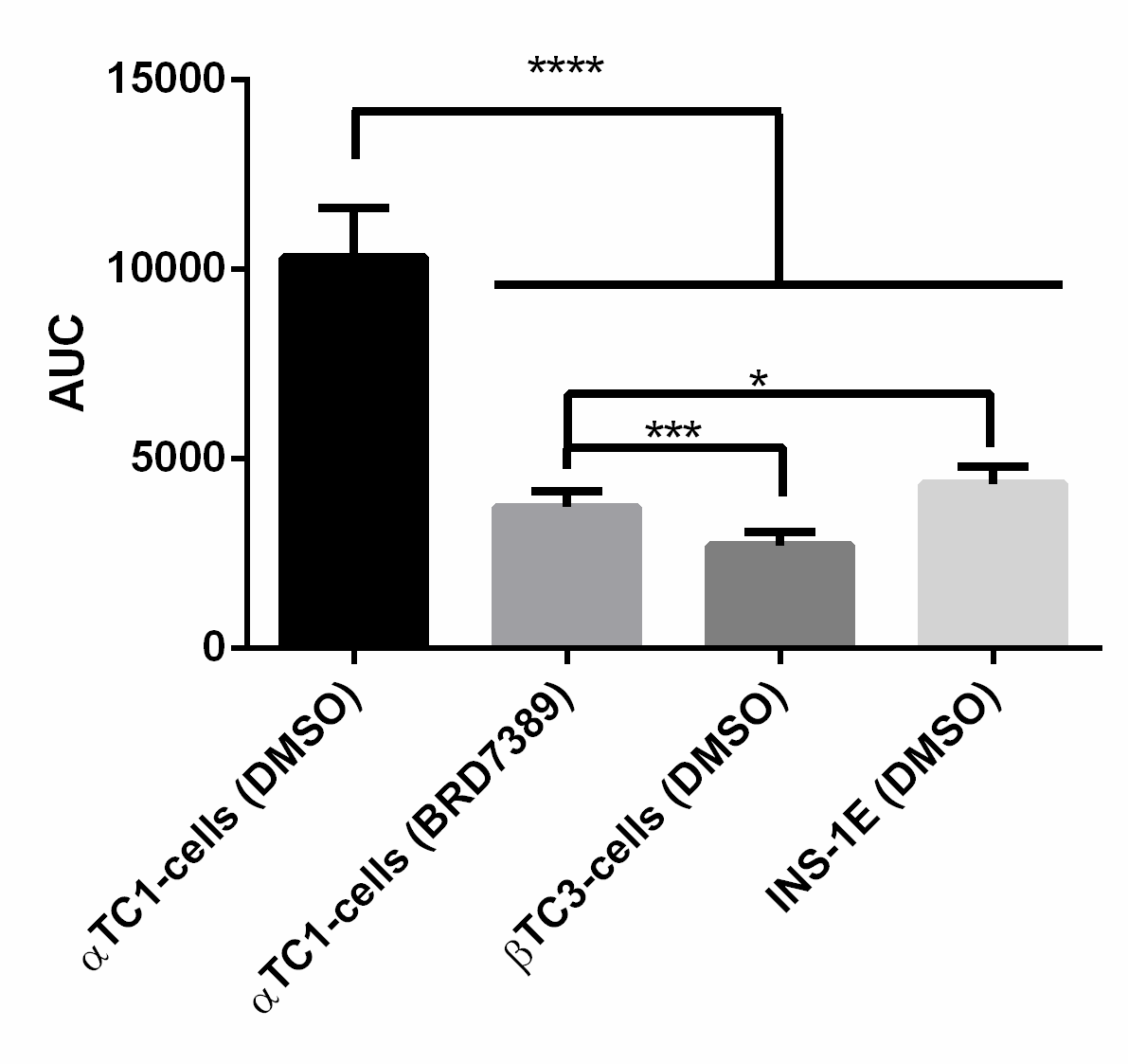** |

**Figure S6.** Gene silencing of Brsk1 and Camkk2. Brsk1 and Camkk2 mRNA levels were assessed after 72-hour transfection with (A) siBrsk1 or (B) siCamkk2. Immunofluorescence detection of Pdx1 in cells treated with (D) siBrsk1, (E) siCamkk2, (F) STO-609, (G) DMSO, (H) KN62, or (I) KN93. Scale bars = 50 µm. Knockdown of (J) BRSK1 (K) CAMKK2

| 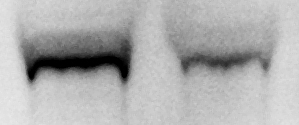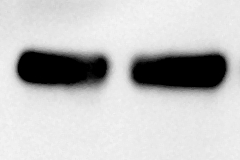**J**  siBrsk1  scramble  BRSK1  β-Actin | 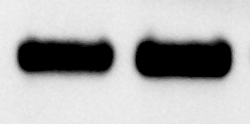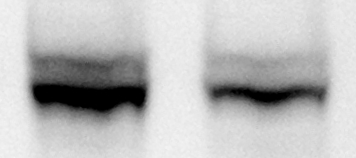**K**  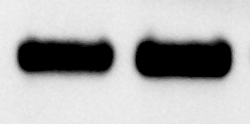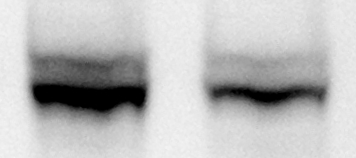  siCamkk2  scramble  CAMKK2  β-Actin |
| --- | --- |

**Table S1.** shRNA hairpins used in this study

| **Gene** | **Hairpin** |
| --- | --- |
| *Camkk1* | NM_018883.2-2125s21c1  NM_018883.2-1500s21c1  NM_018883.2-1035s21c1  NM_018883.1-1484s1c1  NM_018883.2-781s21c1 |
| *Brsk1* | NM_001003920.2-444s21c1  XM_356021.1-484s1c1  XM_356021.1-72s1c1  XM_356021.1-1157s1c1 |
| *STK11* | NM_011492.3-208s21c1  NM_011492.1-1585s1c1  NM_011492.3-854s21c4  NM_011492.3-1250s21c1  NM_011492.1-1189s1c1 |
| *Camk4* | NM_009793.3-1746s21c1  NM_009793.3-437s21c1  NM_009793.3-2218s21c1  NM_009793.1-765s1c1  NM_009793.1-897s1c1 |
| *Camkk2* | NM_145358.1-1043s1c1  NM_145358.1-178s1c1  NM_145358.1-178s21c1  NM_145358.1-1043s21c1  NM_145358.1-1516s1c1 |
| *Camk1* | NM_133926.1-309s1c1  NM_133926.2-790s21c1  NM_133926.1-865s1c1  NM_133926.2-309s21c1  NM_133926.1-790s1c1 |
| Controls | rfp_401s1c1  lacZ_1758s1c1  P2tG-nullT |

**Table S2. Primers sets used for qPCR detection of gene expression of targets of interest. Left primer = forward, right primer = reverse.**

| ***Gene*** | ***Primer set*** |
| --- | --- |
| ***ActB*** | GGTGGGAATGGGTCAGAAGGAC/GGCCACACGCAGCTCATTGT |
| ***Arx*** | TCCAACCCTCCAGGAGAGAGG/CAGCTCAGCCTCGAACGGGG |
| ***Brn4*** | CGTGTTCTCGCAGACTACCA/CAGTACGCCCTTGACACTCA |
| ***Gapdh*** | CTTGTCATCAACGGGAAGCCC/TGACCCTTTTGGCTCCACCC |
| ***Gcg*** | ATCATTCCCAGCTTCCCAGA/CGGTTCCTCTTGGTGTTCAT |
| ***GK*** | TGGATGACAGAGCCAGGATGG/ACTTCTGAGCCTTCTGGGGTG |
| ***Glut2*** | CGGTGGGACTTGTGCTGCTGG/CTCTGAAGACGCCAGGAATTCCAT |
| ***Ins2*** | TTTGTCAAGCAGCACCTTTG/GCAGCACAGAAGCAAAGACA |
| ***MafA*** | ATCATCACTCTGCCCACCAT/AGTCGGATGACCTCCTCCTT |
| ***MafB*** | CAACAGCTACCCACTAGCCA/GGCGAGTTTCTCGCACTTGA |
| ***NeuroD*** | CTTGGCCAAGAACTACATCTGG/GGAGTAGGGATGCACCGGGAA |
| ***Ngn3*** | CTGCGCATAGCGGACCACAGCTTC/CTTCACAAGAAGTCTGAGAACACCAC |
| ***Nkx1.6*** | TCTTCTGGCCTGGGGTGATG/GTGCTTCTTTCTCCACTTGGTCC |
| ***Nkx2.2*** | CATCTTGGACCTTCCGGACAC/GGACTTGGAGCTCGAGTCTTG |
| ***Pax4*** | GACGCTACTACCGCACAGGT/AGCCTTGTCCTGGGTACAAA |
| ***Pax6*** | TCACAGCGGAGTGAATCAGCT/TACTCACAACCGTTGGATAC |
| ***Pdx1*** | ACCACCTTCCAGCTCAGCTCC/CGGGTGTAGGCAGTACGGGTC |

**Table S3.** Gene sets enriched in alpha cells

| NAME | SIZE | ES | NES | NOM p-val | FDR q-val | FWER p-val |
| --- | --- | --- | --- | --- | --- | --- |
| Leonard_Hypoxia | 28 | 0.7340 | 2.0864 | 0 | 0.0009 | 0.001 |
| Mootha_Voxphos | 75 | 0.6132 | 2.0801 | 0 | 0.0005 | 0.001 |
| Siligan_Bound_By_Ews_Flt1_Fusion | 30 | 0.7408 | 2.0471 | 0 | 0.0024 | 0.008 |
| Reactome_Electron_Transport_Chain | 59 | 0.6026 | 1.9817 | 0 | 0.0078 | 0.034 |
| Mootha_Glycolysis | 19 | 0.7631 | 1.9617 | 0 | 0.0107 | 0.055 |
| Kegg_Rna_Degradation | 42 | 0.6379 | 1.9580 | 0 | 0.0094 | 0.058 |
| Reactome_Glycolysis | 17 | 0.7753 | 1.9257 | 0 | 0.0158 | 0.109 |
| Reactome_Branched_  Chain_Amino_Acid_Catabolism | 16 | 0.7957 | 1.9069 | 0 | 0.0194 | 0.152 |

**Table S4.** Gene sets enriched in beta cells.

| NAME | SIZE | ES | NES | NOM p-val | FDR q-val | FWER p-val |
| --- | --- | --- | --- | --- | --- | --- |
| Mor_Plasma_Cell_Up | 30 | -0.79166 | -2.20545 | 0 | 0.0000 | 0.000 |
| Kim_Response_To_TSA_And_Decitabine_Up | 87 | -0.62113 | -2.10434 | 0 | 0.0017 | 0.003 |
| Kegg_Lysosome | 99 | -0.59135 | -2.01935 | 0 | 0.0063 | 0.017 |
| Ren_Alveolar_Rhabdomyosarcoma_Dn | 356 | -0.50624 | -1.99889 | 0 | 0.0055 | 0.020 |
| Mishra_Carcinoma_Associated_Fibroblast_Dn | 18 | -0.80431 | -1.98008 | 0 | 0.0069 | 0.031 |
| Kegg_Antigen_Processing_And_Presentation | 43 | -0.66277 | -1.96686 | 0 | 0.0088 | 0.047 |
| Grade_Colon_And_Rectal_Cancer_Dn | 60 | -0.59983 | -1.91108 | 0 | 0.0224 | 0.136 |

**Table S5. Decreased phosphorylation in S6 and EF2 upon treatment of αTC1 cells with BRD7389 or GW8510 treatment**.

| Compound treatment | IPI_phosphosite_number of sites on peptide | Protein name | phosphosite ratio (log2) | *p*-value |
| --- | --- | --- | --- | --- |
| BRD7389/DMSO | IPI00113655_240_4 | S6 | -0.36 | 0.026 |
| BRD7389/DMSO | IPI00113655_247_4 | S6 | -0.36 | 0.026 |
| BRD7389/DMSO | IPI00113655_244_3 | S6 | -0.34 | 0.033 |
| BRD7389/DMSO | IPI00113655_240_2 | S6 | -0.19 | 0.152 |
|  |  |  |  |  |
| GW8510/DMSO | IPI00466069_57_1 | EF2 | -1.54 | 0.020 |
| GW8510/DMSO | IPI00466069_502_1 | EF2 | -1.41 | 0.032 |

**Table S6.** Phosphosite ratios of Brsk1 and Camkk2 in αTC1 cells, βTC3cells, and compound-treated αTC1 cells.

| IPI_phosphosite_number of sites on peptide | Protein name | Median βTC3/αTC1 phosphosite ratio (log2) | BRD7389/DMSO treatment, 5 day (log2) | GW8510/DMSO treatment, 5 day (log2) |
| --- | --- | --- | --- | --- |
| IPI00515701_434_1 | Brsk1 | -1.15 | -1.27 | nd |
| IPI00515701_583_3 | Brsk1 | -2.81 | -0.64 | -1.48 |
| IPI00515701_587_3 | Brsk1 | -2.81 | -0.64 | -1.48 |
| IPI00515701_508_1 | Brsk1 | -0.71 | nd | -1.19 |
| IPI00480226_495_1 | Camkk2 | -1.85 | -0.52 | -0.62 |
